# Displasia septo-óptica plus: reporte de caso para revisar y reconocer esta entidad

**DOI:** 10.7705/biomedica.7370

**Published:** 2024-11-06

**Authors:** Alexander Reyes, Julieth Galvis, Yilver Estupiñán

**Affiliations:** 1 Grupo para la Renovación Educativa de Medicina Interna (GERMINA), Facultad de Salud, Universidad Industrial de Santander, Bucaramanga, Colombia Universidad Industrial de Santander Facultad de Salud Universidad Industrial de Santander Bucaramanga Colombia; 2 Servicio de Radiología, Hospital Internacional de Colombia, Bucaramanga, Colombia Hospital Internacional de Colombia Bucaramanga Colombia; 3 Servicio de Hospitalización, Hospital Internacional de Colombia, Bucaramanga, Colombia Hospital Internacional de Colombia Bucaramanga Colombia

**Keywords:** displasia septo-óptica, tabique pelúcido, hipoplasia del nervio óptico, imagen de difusión por resonancia magnética, trastornos del neurodesarrollo, epilepsia, Septo-optic dysplasia, septum pellucidum, optic nerve hypoplasia, diffusion magnetic resonance imaging, neurodevelopmental disorders, epilepsy

## Abstract

La displasia septo-óptica es una afección neurológica congénita de etiología multifactorial caracterizada por agenesia del *septum pellucidum,* disgenesia del cuerpo calloso o ambas, hipoplasia del quiasma o nervios ópticos y disfunción hormonal con alteraciones hipofisiarias o hipotalámicas. Para hacer el diagnóstico se requieren dos de estos criterios y la resonancia magnética es el examen de elección. La mayoría de los casos se presentan con anomalías del desarrollo cortical en la forma conocida como displasia septo-óptica plus. Si bien las convulsiones y los trastornos del neurodesarrollo son las manifestaciones neurológicas dominantes, es una entidad muy heterogénea con múltiples hallazgos clínicos y radiológicos que se deben considerar. Se presenta el caso de un hombre de 35 años con antecedentes de traumatismo craneoencefálico en la infancia y remisión por epilepsia focal resistente al tratamiento asociada con déficit cognitivo.

En la evaluación inicial, la tomografía computarizada de cráneo simple se apreció agenesia del *septum pellucidum* y disgenesia del cuerpo calloso. Se practicó una resonancia magnética cerebral que confirmó la agenesia del *septum pellucidum* y, también, reveló irregularidad y engrosamiento anómalo de la corteza cerebral en los lóbulos frontales y la región perisilviana; además, se encontró sustancia gris heterotópica en los lóbulos frontales y la región frontoinsular izquierda, leve ventriculomegalia supratentorial, apariencia atípica del *rostrum* del cuerpo calloso e hipoplasia del quiasma y los nervios ópticos. Aunque la agenesia del *septum pellucidum* fue la clave en este caso, no es un hallazgo presente en todos los pacientes. La relevancia de la resonancia magnética para evaluar detalladamente otras estructuras involucradas, destacándose el nervio óptico hipoplásico, es fundamental en el trabajo diagnóstico del radiólogo y el reconocimiento de esta entidad.

## Abstract (en)

Septo-optic dysplasia is a congenital neurological condition with multifactorial etiology, characterized by *septum pellucidum* agenesis and/or corpus callosum dysgenesis, hypoplasia of the chiasm or optic nerves, and hormonal dysfunction with pituitary or hypothalamic alterations. Diagnosis requires two of these criteria and magnetic resonance is the imaging test of choice. Most cases present with abnormalities of cortical development in the form known as septo-optic dysplasia plus. While seizures and neurodevelopmental disorders are the dominant neurological manifestations, this entity is highly heterogeneous and has multiple clinical and radiological findings to consider.

We present the case of a 35-year-old man with a history of cranioencephalic trauma in childhood and remission for refractory focal epilepsy associated with cognitive deficit. During the initial examination, the simple cranial tomography showed *septum pellucidum* agenesis and corpus callosum dysgenesis. Brain magnetic resonance imaging revealed agenesis of the *septum pellucidum,* irregularity and anomalous thickening of the cerebral cortex in frontal lobes and perisylvian region, heterotopic gray matter in frontal lobes and left fronto-insular region, mild supratentorial ventriculomegaly, atypical appearance of the corpus callosum rostrum, and hypoplasia of the chiasm and optic nerves. Although agenesis of the *septum pellucidum* was the key finding in this case, it is not present in all patients. The relevance of magnetic resonance imaging for the detailed evaluation of other involved structures, highlighting optic nerve hypoplasia, is fundamental in the radiologist's diagnostic workup and this entity recognition.

La displasia septo-óptica, también conocida como el síndrome de De Morsier, es una afección neurológica congénita poco común caracterizada por una tríada conformada por el desarrollo anormal de las estructuras de la línea media, como la agenesia del *septum pellucidum* o la disgenesia del cuerpo calloso, la hipoplasia del quiasma o los nervios ópticos y la disfunción hormonal con alteraciones hipofisiarias o hipotalámicas. Para la confirmación diagnóstica, se requiere la presencia, al menos, de dos de los criterios mencionados. Aunque esta condición no es completamente desconocida, aún existen algunas controversias sobre ella y muchos especialistas - radiólogos, pediatras y neurólogos- deberían incluirla rutinariamente en su diagnóstico diferencial al evaluar pacientes con síntomas neurológicos o epilepsia.

El diagnóstico de displasia septo-óptica puede ser un desafío, ya que tiene un amplio espectro de hallazgos clínicos y radiológicos ^1^ y es muy heterogénea clínicamente, con una variabilidad significativa en cuanto a la gravedad y el tipo de manifestaciones neurológicas. Debido a esto, algunos investigadores han postulado que, más que una condición única, es un síndrome ^2^.

Algunos estudios han informado que la tríada completa está presente solo del 30 al 47 % de los pacientes diagnosticados. En otros trabajos, el 62 % de los enfermos puede presentar hipopituitarismo, el 60 %, ausencia del *septum* pellucidum, y alrededor del 70 % tiene hipoplasia de las vías ópticas, segmentaria o parcial, e incluso, unilateral o bilateral.

Considerando esta variabilidad y la coexistencia de otros hallazgos, algunos autores han considerado la displasia septo-óptica, más que una enfermedad, un espectro de malformaciones, utilizándose en algunos casos el término DSO PLUS (DSO +) ^2^. El término DSO-plus fue acuñado por Miller *et al.* en el 2000 ^3^ para aquellos casos en los que, además de los hallazgos típicos de la displasia septo-óptica, también se presentaban trastornos del neurodesarrollo y malformaciones del desarrollo cortical, como polimicrogiria, esquizencefalia, heterotopías de sustancia gris y displasia cortical. En consecuencia, un caso de displasia septo-óptica con anomalías del desarrollo de la corteza cerebral, se consideraría como DSO-plus. Paradójicamente esta última entidad es más frecuente que la forma original.

Desde su descripción inicial por George de Morsier en 1957 ^3^, los estudios sobre la displasia septo-óptica han asociado su etiología con diversos agentes. La lista incluye infecciones por virus como el citomegalovirus, lesiones vasculares, mutaciones genéticas, edad materna, diabetes gestacional y sustancias tóxicas como el alcohol, así como algunos medicamentos antiepilépticos o la quinidina.

La mayoría de los casos son esporádicos. Los avances en investigación genética han llevado al descubrimiento de mutaciones que podrían estar ligadas a esta entidad, dados los aspectos embriológicos comunes entre la vía óptica y la hipófisis.

Los genes originalmente relacionados con la displasia septo-óptica son el *HESX1* y el *SOX2,* y posteriormente el *SOX3.* No obstante, un número cada vez mayor de genes, como el *OTX2* y el *PROKR2,* ha sido implicado en su etiología, además de factores de transcripción como los de crecimiento de fibroblastos *(fibroblastgrowth factor,* FGF), FGF1 y FGF8 ^4^^,^^5^. Dada esta variabilidad genética, el fenotipo y las manifestaciones radiológicas varían significativamente entre los individuos, incluso entre gemelos.

El gen *HESX1* quizás sea el más estudiado. Este es un gen con dominio *homeobox,* que actúa principalmente como un represor de la transcripción y es uno de los primeros marcadores del desarrollo de la hipófisis en múridos.

Se han identificado mutaciones del genSOX2 asociadas con anomalías oculares bilaterales graves, defectos del cuerpo calloso e hipoplasia hipofisaria. Además, se han descrito otras características, como retraso en el desarrollo, anomalías del aparato genital masculino, atresia esofágica y pérdida auditiva neurosensorial. Los genes *SOX2* y *SOX3* están relacionados con otros trastornos como holoprosencefalia, microftalmia o anoftalmia.

A pesar del descubrimiento de estos genes y su asociación con la displasia septo-óptica, se estima que en menos del 1 % de los casos se ha confirmado que los pacientes son portadores de sus mutaciones patogénicas. Por lo tanto, es importante considerar los otros factores implicados -ya mencionados anteriormente-en el origen de esta compleja condición. De hecho, se ha establecido una clara asociación entre el consumo de alcohol y las malformaciones de las estructuras de la línea media cerebral.

## Presentación del caso

Se trata de un hombre de 38 años, remitido al Hospital Internacional de Colombia (Bucaramanga) por epilepsia focal resistente al tratamiento asociada con déficit cognitivo.

Como antecedentes relevantes, el paciente fue producto único del tercer embarazo, nacido por parto vaginal sin complicaciones, con neurodesarrollo normal hasta los 10 años y sin antecedentes de neuroinfecciones, ni cirugías mayores o trastornos hematológicos. Refirió haber sufrido un trauma craneoencefálico a los 10 años, después del cual desarrolló un aparente deterioro cognitivo con mal rendimiento escolar. Un año después del trauma, presentó su primera crisis convulsiva. Las crisis consistían en movimientos clónicos en el hemicuerpo izquierdo, con mayor afección del miembro superior y, en ocasiones, se acompañaban de cefalea. En el trazado electroencefalográfico, se observó actividad interictal e ictal en la región fronto-central derecha.

Se le ordenaron estudios de laboratorio para descartar trastornos infecciosos y metabólicos, los cuales resultaron negativos. Se le practicó una tomografía computarizada simple de cráneo en la que se observó agenesia del *septum pellucidum* y disgenesia del cuerpo calloso.

**Figura 1:**
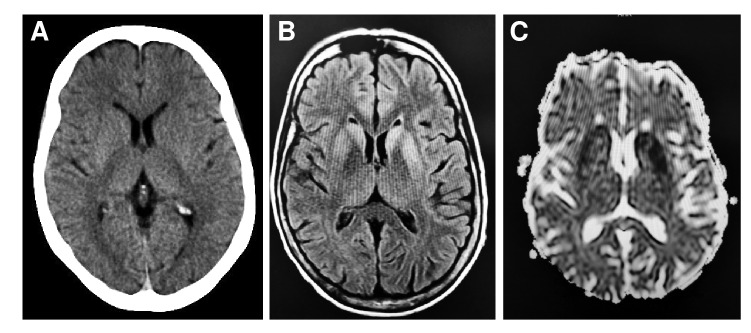
Resonancia magnética: **A)** Corte axial potenciado en T_1_. En el lóbulo frontal derecho, se observa una banda de sustancia gris heterotópica, anormal e irregular, que se extiende desde la corteza hasta el ventrículo lateral derecho. El *septum pellucidum* está ausente y existe una leve ventriculomegalia. **B)** Corte coronal ponderado para T_1_. Se visualiza la hipoplasia del quiasma óptico. Se observa, también, engrosamiento anormal de la sustancia gris en la región medial de ambos lóbulos frontales, indicativa de polimicrogiria bilateral. **C)** Corte axial en T_2_ a la altura de los ventrículos laterales. Se observa agenesia del *septum pellucidum,* ambos ventrículos laterales aparecen unidos y presentan ligera dilatación. En la región frontal derecha, se aprecia una zona focal isointensa con la corteza cerebral, la cual se extiende desde la superficie de la pía hasta la pared ventricular, asociada con heterotopia cortical.

Posteriormente, se complementó con una resonancia magnética cerebral con el protocolo para epilepsia, en la cual se apreciaron varios hallazgos clínicamente relevantes, como la confirmación de la agenesia del *septum pellucidum,* y sugestivos de polimicrogiria, como engrosamiento anormal e irregularidad en varias zonas de la corteza cerebral, particularmente en los lóbulos frontales y en ambas regiones perisilvianas. Además, se identificó sustancia gris heterotópica en los lóbulos frontales, más notoria en el derecho, donde se identificaron dos bandas de sustancia gris anormal y engrosada que se extendían desde la corteza hasta la pared del ventrículo lateral (figuras 1 y 2).

**Figura 2:**
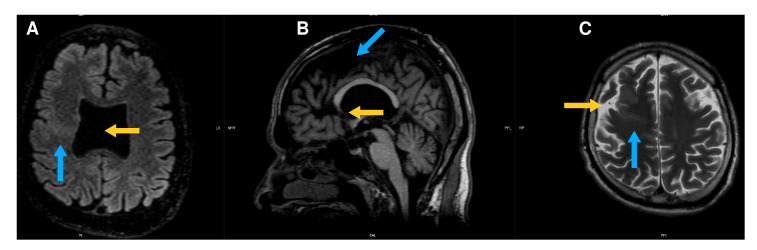
Hallazgos en la resonancia magnética de recuperación de la inversión atenuada de fluido (FLAIR). **A)** Corte axial potenciado: se observa sustancia gris anormal en el lóbulo frontal derecho, la cual se extiende desde la periferia hasta la pared del ventricular. No se visualiza el *septum pellucidum.* **B)** Corte sagital potenciado en T_1_: el cuerpo calloso está presente y el *rostrum* tiene aspecto atípico. Además, se observa prominencia del espacio subaracnoideo en un rango anormal para la edad del paciente, lo que sugiere cambios atróficos. **C)** Corte axial ponderado en T_2_ a nivel frontoparietal superior: en el lóbulo frontal derecho, se observan dos bandas de sustancia gris irregular y heterotópica que se extiende a zonas subcorticales. Se aprecia prominencia del espacio subaracnoideo.

En la región fronto-insular izquierda, se identificó otra banda de sustancia gris heterotópica, paralela al ventrículo lateral. Asimismo, se encontró ventriculomegalia supratentorial y, aunque el cuerpo calloso estaba presente, su pico o *rostrum* tenía una apariencia atípica. El quiasma y los nervios ópticos eran hipoplásicos (figura 1).

Cabe anotar que los hallazgos en el electroencefalograma se correlacionaron con zonas de polimicrogiria y heterotopia cortical en el hemisferio cerebral derecho.

Aunque existía el antecedente de un trauma craneoencefálico, ante la ausencia de hallazgos como zonas de gliosis o sangrados, se consideró que dicho trauma fue leve, sin secuelas estructurales evidenciables en los dos estudios obtenidos. Por ende, lo más probable es que este antecedente no tuviera relación directa con las manifestaciones clínicas del paciente.

Una vez confirmado el diagnóstico, ante el control inicial de los síntomas, y dada la presencia de lesiones bilaterales que comprometían algunas áreas críticas, se decidió brindar tratamiento médico con un nuevo esquema de medicamentos anticonvulsivos y hacer seguimiento periódico en la institución.

### Diagnóstico

Dado el amplio espectro de manifestaciones clínicas y radiológicas de la displasia septo-óptica, el papel del radiólogo es crucial para lograr un diagnóstico preciso, especialmente, teniendo en cuenta que dos de los tres criterios para el diagnóstico se basan en imágenes diagnósticas (agenesia del *septum pellucidum* o disgenesia del cuerpo calloso, e hipoplasia de las vías ópticas). El primer examen de imágenes suele ser la tomografía computarizada de cerebro, la cual puede demostrar la ausencia del *septum pellucidum,* pero no permite evaluar adecuadamente el quiasma y los nervios ópticos. Se requiere, entonces, de la resonancia magnética, cuya interpretación adecuada es esencial, destacándose que esta modalidad también permite visualizar la glándula hipófisis y las vías ópticas.

En 1989, Barkovich *et al.* resaltaron la importancia de identificar la agenesia del *septum pellucidum,* dada su asociación con la displasia septo-óptica y con otras entidades como la esquizencefalia. Estos autores desarrollaron inicialmente el concepto de displasia septo-óptica-plus, al considerar la existencia de dos subgrupos de pacientes: unos con esquizencefalia y otros sin ella ^6^.

Según lo anterior y desde el punto de vista radiológico, quizás la pista más importante para el diagnóstico de displasia septo-óptica sea la ausencia del *septum pellucidum,* ya que este puede ser el único hallazgo ^7^ y es fácilmente identificable. Sin embargo, esta agenesia no siempre se presenta ^5^, por lo que se requiere un análisis minucioso de todas las imágenes, que incluyan la evaluación de las vías ópticas y la glándula hipófisis.

Sin lugar a duda, el examen de imágenes diagnósticas de referencia es la resonancia magnética. Las imágenes sagitales potenciadas en T_1_ o T_2_ permiten visualizar el cuerpo calloso y los nervios ópticos, y en conjunto, los cortes coronales y axiales muestran adecuadamente el sistema ventricular, el *septum pellucidum* y el quiasma óptico. Los globos oculares también pueden evaluarse, particularmente, en las imágenes axiales potenciadas en T_2_.

Otros hallazgos potenciales son microftalmia, anoftalmia, coloboma, hipoplasia o ectopia hipofisaria, e hipoplasia del tallo hipofisario; además, otros descritos más recientemente, como la reducción de los diámetros del mesencéfalo, el bulbo, la protuberancia y el tracto olfatorio ^4^.

### Vías ópticas y septum pellucidum

Los radiólogos y los médicos tratantes deben familiarizarse con la valoración de las estructuras ópticas. En la literatura existen publicaciones con aproximaciones sobre las medidas normales de los nervios y el quiasma óptico en población adulta. Mncube y Goodier ^8^ encontraron que el ancho normal del quiasma óptico está entre 11,13 y 16,92 mm, con una media de 13,63 mm.

Otro estudio realizado por Wagner *et al.*^9^, informó que, en las imágenes coronales de la resonancia magnética, el quiasma óptico tiene un área entre 33,3 y 54,1 mm^2^ y un ancho entre 10,6 y 17,4 mm. Para los autores de este último estudio, los valores fuera de este rango pueden considerarse anormales.

Por lo tanto, durante el análisis de las imágenes, se puede medir el quiasma para confirmar si hay hipoplasia ^10^.

En una publicación reciente de 48 pacientes ^11^, se encontró que 47 de ellos presentaban hipoplasia del quiasma o de los nervios ópticos, unilateral o bilateral. Además, se encontró agenesia del *septum pellucidum* en prácticamente todos los pacientes y el 47 % de estos tenía hipoplasia hipofisiaria.

Por lo tanto, la evaluación adecuada de las vías ópticas, del *septum pellucidum* y de la glándula hipófisis, es crucial en esta condición.

## Displasia septo-óptica plus

La displasia septo-óptica tiene presentaciones muy variables. Además de los hallazgos clásicos descritos, existen otras características potenciales que pueden asociarse con esta condición ^6^^,^^12^^,^^13^. En el 2000, Miller *et al.*^12^ propusieron el nombre de displasia septo-óptica plus para aquellos casos que también presentaban retraso del neurodesarrollo, malformaciones del desarrollo cortical y esquizencefalia; está última es la más frecuente ^6^^,^^12^^,^^13^^,^^15^.

En una publicación en la que se analizaron seis pacientes adultos con displasia septo-óptica y epilepsia farmacorresistente ^16^, se demostró que todos ellos presentaban malformaciones corticales como causa de las convulsiones. En un estudio más amplio, en el cual se incluyeron 17 pacientes con displasia septo-óptica, solo en uno (6 %) la enfermedad se clasificó como displasia septo-óptica clásica, mientras que en 13 (76 %), se clasificó como displasia septo-óptica plus. En este mismo estudio, solo en 6 (18 %) pacientes el *septum pellucidum* era normal ^17^.

Ward *et al.* incluyeron un mayor número de pacientes y hallaron una prevalencia del 56 % de malformaciones corticales en aquellos con diagnóstico de displasia septo-óptica ^12^. En conjunto, lo anterior indica que es más probable hacer el diagnóstico de displasia septo-óptica plus que el de la forma clásica de displasia septo-óptica.

Las malformaciones corticales son anomalías macroscópicas o microscópicas de la corteza cerebral, las cuales se originan por interrupción del proceso normal de formación cortical, por lo que se requiere una evaluación meticulosa de la corteza cerebral. Estos trastornos son causas comunes de retraso en el desarrollo neurológico y de epilepsia ^18^^,^^19^.

En el cuadro 1, se presentan las características esenciales de las cuatro condiciones que tradicionalmente se han relacionado con la displasia septo-óptica.

**Cuadro 1:** Principales características de las malformaciones del desarrollo cortical asociadas con la displasia septo-óptica

Trastorno	Principales hallazgos
Esquizencefalia	Hendidura transcortical revestida por sustancia gris que se extiende desde la superficie de la pía hasta la pared ventricular.
Polimicrogiria	Corteza anormal, engrosada e irregular. La región afectada presenta contornos lobulados, en consecuencia, la transición cortical-subcortical no es lisa sino aparentemente lobulada.
Heterotopia de la sustancia gris	Bandas o nódulos de sustancia gris (que siguen la intensidad de señal de la corteza en las diferentes secuencias), situados en una posición anormal, adyacentes a la pared ventricular o en regiones subcorticales.
Displasia cortical focal	Presentación variable. Engrosamiento cortical focal con intensidad de señal que puede ser anormal. Signo de transmanto. Alteración de la diferenciación córtico-subcortical. Existen tres formas de esta afección.

Reproducida y traducida con autorización de J Radiol Med Imaging. 2021:4(1);1044

### Otras formas de la enfermedad

El retraso en el desarrollo neurológico es una condición muy prevalente entre los pacientes con esta condición, con una proporción incluso del 78 % en una serie ^17^. En múltiples trabajos se ha reportado también hidrocefalia, por lo que es posible que una mayor proporción de los afectados presente reducción del volumen cerebral o atrofia; así fue en el presente caso, en el cual se presentó ventriculomegalia leve y aumento en la amplitud del espacio subaracnoideo, con poca reducción del volumen cerebral (figura 2).

Las convulsiones y el retraso intelectual son los síntomas neurológicos dominantes. No obstante, otras manifestaciones menos comunes también pueden sugerir este diagnóstico, por ejemplo, hipoglucemia, espasmos infantiles, midriasis congénita, hipoplasia del bulbo olfatorio, talla baja, microftalmia compleja y obesidad infantil ^5^^,^^20^^-^^24^.

## Abordaje diagnóstico

**Figura 3:**
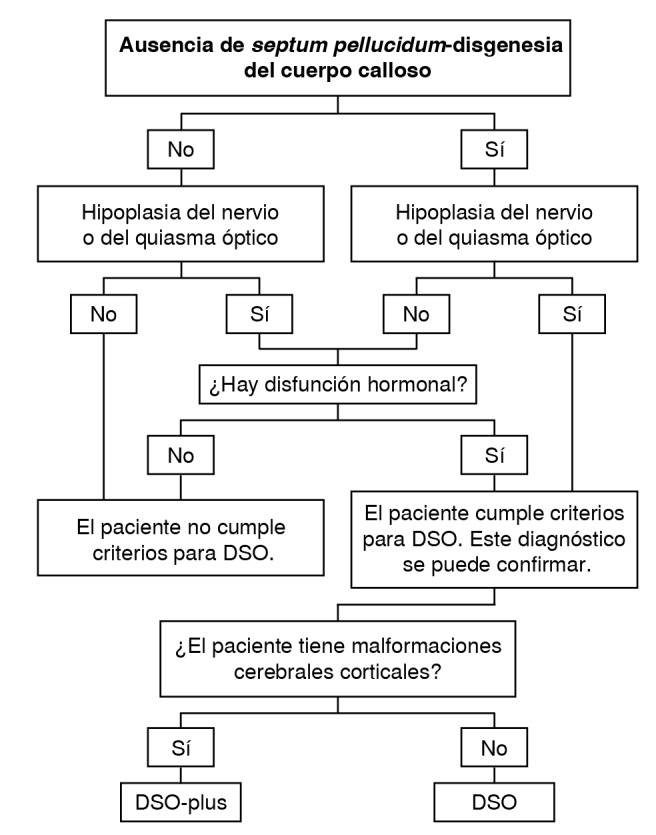
Algoritmo diagnóstico sugerido para evaluar la displasia septo-óptica

En este trabajo se propone un algoritmo (figura 3) basado en imágenes por resonancia magnética ^25^^)^ para el diagnóstico de la displasia septo-óptica (figura 3). Se inicia con la evaluación del *septum pellucidum* y, si se observa agenesia, se debe descartar una posible hipoplasia de la vía óptica o de la glándula hipófisis. En este punto, se confirmaría el diagnóstico con dos de los tres criterios diagnósticos. En caso de encontarse solo una de estas alteraciones, se deben solicitar pruebas de laboratorio para estudiar la función hormonal.

DSO: displasia septo-óptica

Reproducida y traducida con autorización de J Radiol Med Imaging. 2021:4(1);1044

## Conclusiones

La displasia septo-óptica puede adoptar diversas formas, tanto en sus manifestaciones clínicas como en el estudio radiológico. Se puede encontrar casos graves que se diagnostican en la primera infancia, hasta adultos con la epilepsia como única manifestación. No obstante, en cualquiera de estas circuntancias, las imágenes diagnósticas son esenciales para un diagnóstico preciso y oportuno.

Hoy en día, se considera que la etiología de la displasia septo-óptica es multifactorial. Aunque varias mutaciones genéticas se han vinculado con esta entidad, la mayoría de los casos son esporádicos y se pueden asociar con lesiones vasculares, o con otros factores tóxicos o ambientales.

La triada diagnóstica clásica para la displasia septo-óptica está conformada por disgenesia del *septum pellucidum,* del cuerpo calloso o de ambos, por hipoplasia de las vías ópticas y por disfunción hormonal. Se requieren dos de estos tres criterios para hacer el diagnóstico.

La displasia septo-óptica plus es un nombre propuesto por Miller *et al.* que se emplea en aquellos casos en los cuales, además de tales hallazgos típicos, se presentan malformaciones del desarrollo cortical.

La resonancia magnética es la modalidad de imagen diagnóstica de referencia en la displasia septo-óptica.
